# Seasonal dynamics of parasitism and stress physiology in wild giant pandas

**DOI:** 10.1093/conphys/coaa085

**Published:** 2020-09-28

**Authors:** Wenliang Zhou, Kai Gao, Yingjie Ma, Le Wang, Meng Wang, Fuwen Wei, Yonggang Nie

**Affiliations:** 1 Key Laboratory of Animal Ecology and Conservation Biology, Institute of Zoology, Chinese Academy of Sciences, Beijing 100101, China; 2 Ministry of Education, Key Laboratory for Biodiversity Science and Ecological Engineering, College of Life Sciences, Beijing Normal University, Beijing 100875, China; 3 University of Chinese Academy of Sciences, Beijing 100049, China; 4 Center for Excellence in Animal Evolution and Genetics, Chinese Academy of Sciences, Kunming 650223, China

**Keywords:** Faecal cortisol levels, giant panda, nutrition, parasites, stress physiology

## Abstract

Many factors, including the inner status of the individuals and external environment, can influence the parasite infections and stress physiology in mammals. Here, we explored the influence of the sex, age, reproductive season and seasonal food availability on the parasitism and stress physiology in wild giant pandas (*Ailuropoda melanoleuca*) through nutrient and steroid hormone analysis and parasitic infection measurement. Diet composition had significant influences on the faecal cortisol levels and parasite load of wild giant pandas. The seasonal dynamic of the cortisol levels and parasite load in faeces co-vary with the seasonal nutrient intake levels of the pandas, which concurrently arrived the peaks at the wood bamboo shoot-eating period in May (parasite infection intensity, 41.47 ± 12.11 eggs/g of wet faeces; cortisol levels, 619.34 ± 70.55 ng/g dry faeces) that the nutrition intake by wild pandas was the highest (protein/fibre, 69.23 ± 9.93). Meanwhile, age class is also as an important factor to affect the parasite load and stress physiology of wild giant pandas. Cubs and sub-adults suffered more helminth burden and stress physiology than adults and old individuals. This is the first study to evaluate the inner and external factors influence on parasitism and stress physiology in wild giant pandas. The findings facilitate a better understanding of how environmental factors might influence the physiology, behaviour and health of pandas and other species and have implications for the conservation and management of the endangered species.

## Teaser

Parasitic infection as a form of chronic stress affects the cortisol metabolite levels, and the cortisol levels were consistent with the seasonal variation in parasite infection. Sex, age, reproductive status and diet composition were directly associated with helminth parasite richness of wild giant pandas. Age and diet composition had significant influences on the faecal cortisol levels.

## Introduction

Nutrition, parasitism and stress hormones have reciprocal effects (Beldomenico and Begon, 2015). It is well known that food shortages or poor-quality diets not only result in malnourishment (Trites and Donnelly, 2003) but also stimulate the host to produce more stress hormones (Muller and Wrangham, 2004; Behie and Pavelka, 2013; Chinn *et al*., 2018). For example, a poor-quality diet corresponded with an increase in cortisol in colobus monkeys (*Procolobus rufomitratus*) (Chapman *et al*., 2007). Most of the previous studies also indicated that poor-quality food could reduce the parasite resistance of the host (Coop and Holmes, 1996; Coop and Kyriazakis, 1999; Agostini *et al*., 2017). Meanwhile, stress hormone levels are largely affected by parasite prevalence or infection intensity (Seguel *et al*., 2019). For instance, the faecal cortisol levels were directly associated with the intestinal parasite richness in wild male chimpanzees (*Pan troglodytes*) (Muehlenbein and Watts, 2010).

In addition to the food availability, the parasite infection of wildlife is also related to body condition (Sánchez *et al*., 2018). Animals in good body conditions might experience an increase in the ability to resist infection by parasites or better tolerate high parasite loads (Roy and Kirchner, 2000). Conversely, poor body condition can result in impaired immunological development, maintenance and function, which could increase susceptibility to infection or lead to high parasite loads and a long infection duration (Beldomenico *et al*., 2008; Beldomenico and Begon, 2010). Meanwhile, faecal cortisol levels are also affected not only by nutrition and parasite infection but also by a variety of other factors, such as sex, age class, body condition, habitat change and diet composition (Girard-Buttoz *et al*., 2009; Pokharel *et al*., 2017; Ranglack *et al*., 2017; Tecot *et al*., 2019). For instance, stress hormones in the plain zebra (*Equus quagga*) in Etosha National Park largely peak in the dry season, when parasite infection intensities are lowest and are most strongly correlated with host mid-gestation rather than with parasite infection intensity (Cizauskas *et al*., 2015). Thus, it is important to concurrently measure multi-factors, such as the sex, age and reproductive season along with their seasonal food fluctuations, influence on the parasitism and stress physiology to better understand their complex interactions.

As an endangered species, the giant panda (*Ailuropoda melanoleuca*) may suffer from a large number of potential stressors, such as habitat reduction, climate change and human disturbance (Zhu *et al*., 2010; Li *et al*., 2015; Wei *et al*., 2015a). However, seasonal diet quality change and potential food competition with sympatric species as potential stressors have been ignored. Previous studies indicated that giant pandas faced nutritional stress in late winter and early spring (from December to next March) when they consumed a less optimal diet during this period (Schaller *et al*., 1985; Li *et al*., 2017; Wei *et al*., 2019). The golden takin (*Budorcas taxicolor bedfordi*) has a broad diet, which includes bamboo leaves and shoots and thus caused competition with the giant panda from June to August in Foping National Nature Reserve (FNNR) (Wei *et al*., 2017). Recent study showed that strong food competition may exist between the giant panda and a sympatric species, the wild boar (*Sus scrofa*), during the bamboo shoot-eating period (mainly from May to July) (Nie *et al*., 2019a). In addition to food-related stresses, the giant panda is also likely to face some threats from disease due to extraintestinal migration (visceral larva migrans) by an ascarid nematode (Zhang *et al*., 2008). The parasite fauna of the wild giant panda consists of five species (*Baylisascaris schroederi*, *Ogmocotyle pygargi*, *Toxascaris seleactis*, *Ancylostoma ailuropodae* n. sp. and *Strongyloides* sp. (larvae)), dominated by *B. schroederi* (Zhang *et al*., 2011). Parasites can cause chronic stress in giant pandas, and the probability of death in wild pandas caused by parasites increased significantly from 1971 to 2005 (Zhang *et al*., 2008).

In this study, we explored the influence of the sex, age, reproductive season and seasonal food availability on the parasitism and stress physiology in wild giant pandas. We used faecal cortisol as a measure of physiological stress in pandas and examined the number of parasite eggs in faecal samples to assess the prevalence and intensity of gastrointestinal helminth infections. Considering all factors influence the parasitism and stress physiology comprehensively, our purpose is to assess the potential influences of environmental factors and individual conditions on the health and survival of wild giant pandas and provide useful information for the conservation and management of such an endangered species.

## Materials and methods

### Study area and sample collection

This study was conducted in FNNR (107°8′E, 33°8′N) in the Qinling Mountains, Shaanxi, China. The reserve was established for the preservation of giant pandas and contains the population with the highest density in the wild (State Forestry Administration, 2015). Two bamboo species, wood bamboo (WB) (*Bashania fargesii*) and arrow bamboo (AB) (*Fargesia qinlingensis*), grow here at mean elevations of 1600 and 2400 m, respectively. As bamboo-eating specialists, the giant pandas in our study showed seasonal foraging migration associated with the phenologies of the two bamboo species (Nie *et al*., 2019b).

**Figure 1:**
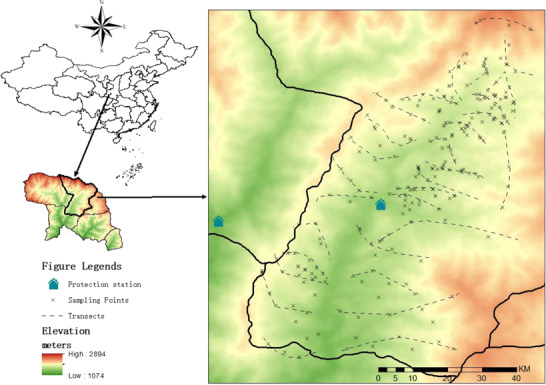
Distribution of transect lines and sampling sites in FNNR, China.

From September 2013 to April 2018, we collected fresh faeces (0–3 h) of giant pandas in the Sanguanmiao Protection Area in FNNR. First, fresh faecal samples were collected by tracking four GPS-collared pandas, two males named Xiyue and Chaoyang, and two females named ZhenZhen and Niuniu, and the son of Niuniu named Longbao, two or three times per week from September 2013 to May 2014. Then, a total of 30 transects were established in our study area with the purpose of fresh faeces collection (Fig. 1), and 10 field staff members were divided into five groups to conduct the sampling work on six consecutive days each month from November 2017 to April 2018. To obtain a sufficient number of samples, we collected all fresh faeces that we encountered during the field work. Each sample was divided into three parts for the individual identification of pandas, parasite analysis and cortisol measures. The samples were stored on ice in 50-ml sterile centrifuge tubes in the field and usually frozen at −30°C within 3 h at the field station. We then transferred the samples with dry ice to the lab at the Institute of Zoology, CAS, until laboratory analysis. Thus, there is no freeze-thaw phenomenon in the transportation of the samples. During all stages of sample collection and processing, we used latex gloves to reduce pollution of the samples.

Bamboo samples were also collected in the same area from September 2013 to August 2014. Samples were collected according to the ages and included one-year- and multi-year-old leaves, culms and shoots (Nie *et al*., 2015). All bamboo samples were oven dried at 55°C and ground to powder with a common multifunctional laboratory mill (KMS-800Y) in the laboratory and then stored at 4°C for subsequent analysis.

### Sex and age determination and individual identification

DNA was extracted from faeces according to Zhang *et al*. (2006) using standard controls. Fifteen microsatellite primers, Ame-μ5, μ10, μ11, μ13, μ15, μ22, μ24, μ26, μ27, Aime1, AY79, AY95, AY161213, AY217 and GP7 (Lu *et al*., 2001; Shen *et al*., 2005; Wu *et al*., 2009; Hu *et al*., 2017), were used to amplify the DNA extracts from the faecal samples. We amplified each extract three times, if the genotype could not be determined, we performed two additional amplifications. Two species-specific sexing primer pairs, SRY and ZF, were designed for sex determination (Zhan *et al*., 2007), which was conducted three times for each DNA extract. Template DNAs from a male, a female and a negative control were amplified in each PCR. Individual and sex identification PCR amplification procedures are described in detail in Hu *et al*. (2010).

The bamboo fragment sizes in the faeces were used to distinguish the age class of giant pandas (Schaller *et al*., 1985). Here, we determined the age class of giant pandas through the bamboo leaves fragments sizes of the faeces. For details, we used a vernier caliper to measure the length and width of bamboo leaves fragments randomly, and no <40 fragments were measured in each faecal sample. The ages of five giant pandas with GPS collars (including a new collared one DianDain) and three offspring (HuZi, HouBao and LongBao) are relatively clear (Table 1). We measured the length and width of bamboo leaves fragments in faeces of these individuals and made the values as the background information for the age estimations of other individuals. Based on this information, all individuals were categorized into one of four age classes: cubs (0–1.5 years), sub-adults (1.5–5.5 years), adults (5.5–20 years) and old (>20 years) (Hu 1987).

**Table 1:** Individual information of giant pandas in the study

Panda ID	Panda name	Sex	Age class (Age estimated)	Infected parasites or not	Samples
S1	XiYue^*^	Male	Adult (17 years)	Yes	70
S2	DianDian^*^	Male	Adult (17 years)	Yes	14
S3	ZhenZhen^*^	Female	Adult (14 years)	Yes	9
S4	HuZi	Male	Adult (7 years)	Yes	4
S5	NiuNiu^*^	Female	Adult (16 years)	Yes	21
S6	LongBao	Male	Cub (1–1.5 years)	Yes	11
S7	ChaoYang^*^	Male	Old (21 years)	Yes	16
S8	HouBao	Male	Cub (1 years)	Yes	1
S9	S9(DG)	Male	Adult	Yes	8
S10	DongDong	Female	Adult	Yes	4
S11	ZLC	Female	Old	Yes	1
S12	LiLi	Female	Sub-adult	Yes	3
S13	DZC	Female	Adult	No	1
S14	LJG	Male	Adult	Yes	7
S15	DZC	Male	Adult	Yes	4
S16	Jiang	Female	Old	No	1
S17	GuGu	Male	Adult	Yes	10
S19	ZWG	Male	Adult	Yes	1
S20	ZW	Female	Adult	Yes	2
S21	DLZG	Male	Old	Yes	6
S22	WFG	Male	Sub-adult	Yes	1
S23	XLZG	Female	Adult	No	1
S24	XLZG	Male	Cub	No	1
S25	XMDG	Female	Adult	No	3
S26	HNB	Male	Sub-adult	No	1
S28	XYP	Female	Adult	No	1
S30	JJG	Female	Sub-adult	Yes	2
S34	LanNi	Female	Adult	No	1
S35	DongYang	Male	Adult	Yes	1
Unknown					98
Total					304

^*^represents the GPS-collared individuals. Because our study spanned a nearly 6-year scale, the age of the giant panda was the age when we collected and measured the bamboo leaves fragment in the faecal samples.

### Nutrient analysis

Through our long-time tracking and foraging behaviour observation on giant pandas, we quantified the diet composition according to the types of bamboos they ingested, for instance, the proportion of one-year and multi-year bamboo leaves in the diet that we recorded. We also calculated the proportion of different bamboo components (e.g. shoots, culms and leaves) in the dung of pandas to confirm the observational result. The macronutrient composition of foods eaten by giant pandas were mainly crude protein, crude fiber, crude fat and carbohydrate. Unlike crude protein and crude fiber, the total crude fat and carbohydrate concentrations were low and did not show significant difference in leaves and shoots of WB and AB (Nie *et al*., 2019b). For the bamboo diet of pandas, protein is thought to be a very important nutrient with high value, which plays key roles in the pandas’ life history (Nie *et al*., 2015; Nie *et al*., 2019b), and the crude protein:fiber ratio can be used to show the food quality of giant panda (Wei *et al*., 2014b). A high-quality diet usually has high protein and low fibre (e.g. young shoots and leaves), thus we use the crude protein:fiber ratio as an index to measure the nutrition intake of giant pandas. We collected faecal samples of giant pandas all years and calculated the proportion of different bamboo components to quantify the diet composition in each month. The quality of the diet in each month was determined by multiplying the proportion of different bamboo components by the protein-to-fibre ratio of each item and summing all products. The total nitrogen content was assessed using Kjeldahl procedures with a Kjeltec™ 8400 analyser. The measurement of total nitrogen provides an estimate of crude protein (protein = N * 6.25). Acid detergent fibre was measured using the methods of Van Soest *et al.* (1991) with a Fibertec™ 2010 Automatic Fiber Determination System.

### Parasites infection analysis

Each fresh faecal sample was examined for parasite eggs using the floatation/microscope technique as described by Zhang *et al*. (2011). Eggs and larvae of parasites were counted and identified based on egg colour, shape, content and size. Prevalence of parasites was determined by dividing the number of positive samples/individuals by the total number of samples/individuals examined. Parasite infection intensity, with uninfected samples being excluded, was defined as the number of conspecific parasites eggs per gram of wet faeces.

### Extraction and mensuration of hormones

Glucocorticoids are an indicator of stress (Möstl and Palme, 2002), and the concentration of hormone metabolites (cortisol) can be measured in animal faeces or other body fluids (Wasser *et al*., 2000; Young *et al*., 2004; Di Francesco *et al*., 2017). For details on the hormone analysis, see Nie *et al*. (2012 a, b), with slight modification in regard to sample pretreatment. We weighed 5–10 g of the fresh faecal samples and then mixed the subsamples and placed them in Petri dishes, and dried the samples for >1 week in a freeze dryer. The freeze-dried faecal samples were crushed with a grinder to ensure that the samples were mixed evenly. We weighed 1 g of the ground faecal samples into a 15-ml centrifuge tube, added 4 ml of an 8:1 mixture of analytically pure methanol and distilled water and mixed and vibrated the tube for 3 min. To remove the lipids, which may impact steroid-antibody binding in the radioimmunoassay, we added 2 ml of analytically pure petroleum ether into the centrifugal tube and vortexed it for 1 min. We rotated the tube in a centrifuge at 10000 rpm for 15 min at −4°C before transferring 2 ml of the methanol layer (the supernatant) to another tube. The supernatant was centrifuged at a speed of 10 000 rpm for 3 min at −4°C, and then 1 ml of solution was added to a new 1.5 ml centrifuge tube, which was cryopreserved at −20°C before analysis. The cortisol concentrations in the supernatant were measured using a commercially available iodinated [I^125^] cortisol radioimmunoassay kit (Jiuding Medical Biological Engineering Co., Ltd, Tianjin, China). A DFM-96 automatic radioimmunoassay r-counter (Zhongcheng Electromechanical Technology Development Co., LTD, Hefei, China) was used to quantify the radioactivity of the supernatant fraction.

### Statistical analyses

For statistical model building, faecal samples were separated into two seasonal groups, the mating season (March–April) and non-mating season (other months), based on the reproductive season of giant pandas (Nie *et al*., 2012b; Zhou *et al*., 2019). Generalised linear mixed-effects models (GLMM) had an advantage in handling non-linear data, which could avoid transformations through the use of a link function, and also non-linear effects between dependent and independent variables. We used GLMM to predict the infection intensity of the parasites and cortisol metabolite concentration in wild giant pandas based on sex (female/male), age (old, adult, sub-adult/cub), reproductive season (mating season/non-mating season) and diet type (bamboo leaves, shoots/culms). Model selection was based on Akaike information criterion corrected (AICc) for small samples, hence models with lowest AICc values were selected as the most parsimonious. Analysis were carried out in R version 3.5.1, using the statistical package lmerTest. Pearson correlation was used to calculate the correlation coefficient (r) of the nutrition (protein/fiber), infection intensity of the parasites and faecal cortisol metabolites. Statistical differences between these factors were determined using the Wilcoxon rank sum test and the Kruskal–Wallis test. All tests were two-tailed tests, with *P* < 0.05 indicating significance. The statistical values are all expressed as the means ± SEM. ArcGIS 10.5 software (Environmental Systems Research Institute Inc., Redlands, USA.) was used to draw a map.

## Results

A total of 304 faecal samples were collected during this study, and 206 of them were identified as originating from 29 giant pandas (13 females and 16 males) (Table 1). A total of 117 faecal samples were collected by tracking the GPS-collared pandas and belonged to five individuals named XiYue, ChaoYang, NiuNiu, ZhenZhen and LongBao from September 2013 to May 2014. The DNA analysis successfully identified 89 samples from 27 individuals (including XiYue, NiuNiu and ZhenZhen) from November 2017 to April 2018, and 22 faecal samples were from 13 females, and 67 faecal samples were from 14 males.

## Seasonal dynamics and correlation of nutrition, parasite infection intensity and cortisol levels

**Figure 2:**
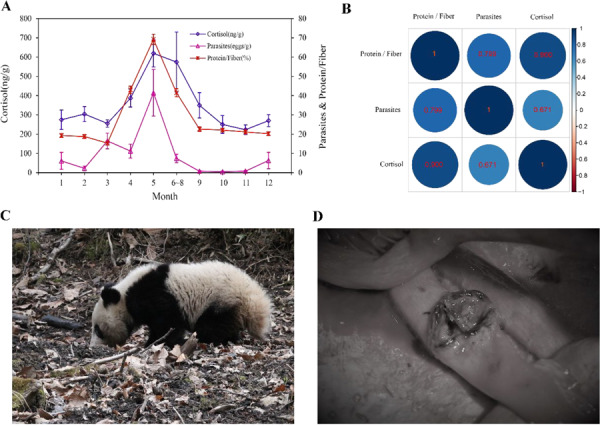
(A) Seasonal variation in the nutrition, infection intensity of the parasites and faecal cortisol metabolites of giant pandas in FNNR. (B) Correlation of the nutrition (protein/fiber), infection intensity of the parasites and faecal cortisol levels. (C) Potential way of parasite infection through polluted water drinking. (D) Intestinal perforation caused by parasitic infection in an old individual died in winter.

The diet nutrients showed obvious seasonal changes (Fig. 2A). Pandas preferred one-year-old WB leaves (protein/fibre %, 22.59 ± 3.90) than multi-year-old WB leaves during the first several months after they move back to the low elevations, usually from September to November (Table 2). As the consumption of one-year WB leaves gradually increased, multi-year WB leaves increased gradually in their daily diet items (protein/fibre %, 17.87 ± 3.08) from December to April of the next year (Table 2). We found that pandas would also feed on a small part of WB culms (protein/fibre %, 2.59 ± 0.35) and AB culms (protein/fibre %, 2.09 ± 0.41) in March (12.3% WB culms and 4.1% AB culms) and April (12.5% WB culms and 7.5% AB culms). In May, the WB shoots were the main food of giant pandas when they were available, and the percent of protein/fibre in the WB shoots (69.23 ± 9.93) was significantly higher than that in the WB leaves. When the WB shoots matured, the percent of protein/fibre decreased, and pandas migrated to the high-elevation site and switched to AB shoots (protein/fibre %, 47.51 ± 7.51) and one-year-old AB leaves (protein/fibre %, 29.92 ± 2.38) from June to August.

**Table 2:** Seasonal variation in diet composition of giant pandas

Month	Jan	Feb	Mar	Apr	May	Jun–Aug	Sep	Oct	Nov	Dec
WB one-year-old leaves	30%	20%				10%	100%	90%	70%	50%
WB multi-year-old leaves	70%	80%	83.6%	27.5%				10%	30%	50%
WB shoots				52.5%	100%					
WB culms			12.3%	12.5%						
AB one-year-old leaves						20%				
AB culms			4.1%	7.5%						
AB shoots						70%				
Protein/fibre (%)	19.29	18.81	15.34	43.04	69.23	41.50	22.60	22.12	21.18	20.23

The seasonal variation of cortisol levels in faeces was similar with the dynamic of parasite infection intensity, which is highly consistent with the seasonal variation of nutrition intake in pandas (Fig. 2A). The intensity of the parasite infection varied in different months and seasons, and the highest value occurred in May (41.47 ± 12.11 eggs/g of wet faeces). From September to November, the parasite infection intensity was very low, among which, it reached the lowest level in October (0.48 ± 0.21 eggs/g of wet faeces) (Fig. 2A). The correlation coefficient between the parasite infection intensity and the nutrition intake of giant pandas was *r* = 0.799 (Fig. 2B). The cortisol levels in the faeces ranged from 33.2 to 1026.8 ng/g, varying in different months and seasons, which rose in April and peaked in May (619.34 ± 70.55 ng/g dry faeces), and went back to low level in September (Fig. 2A). The correlation coefficient between cortisol levels and nutrition intake of giant pandas was *r* = 0.900 (Fig. 2B). Meanwhile, we also found a significant correlation between parasite infection intensity and cortisol levels (*r* = 0.671) (Fig. 2B).

## Parasite prevalence and infection intensity in giant pandas

The overall prevalence of helminth infections was 56.79% (163/287), with 18.43 ± 2.81 eggs/g of wet faeces. Three different eggs of helminth species were identified in the faecal samples. Prevalence of these helminths was not homogenous and *B. schroederi* was dominant, which occupied 96.93% (158/163), followed by *T. seleactis* 1.84% (3/163) and *A. ailuropodae* n. sp. 1.23% (2/163). A total of 72.41% (21/29) of the identified individuals were found to be infected by parasites, and males (87.50%, 15/16) were higher than females (53.85%, 7/13).

**Figure 3:**
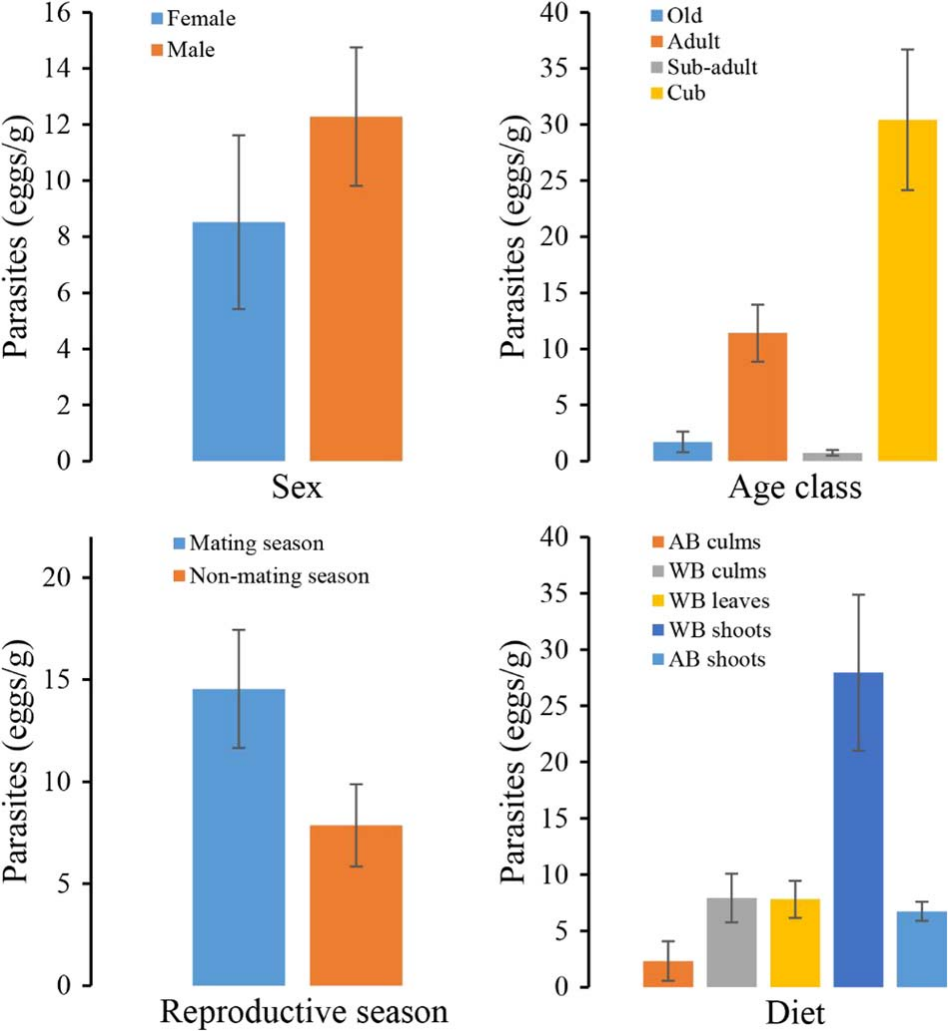
Parasitic infection intensity of giant pandas by sex (A), age (B), reproductive season (C) and diet (D).

Model selection showed that the interaction between reproductive season and diet was the best model (Table 3). Parasite infection intensity was the highest during the WB shoot-feeding period (27.95 ± 6.93 eggs/g wet faeces) than other time (*X*^2^ = 30.009, *P* < 0.0001), which in mating season (14.55.95 ± 2.89 eggs/g wet faeces) was higher than non-mating season (7.86 ± 2.02 eggs/g wet faeces) (*W* = 6708.5, *P* < 0.0001) (Fig. 3C, 3D). Although age class was not included in the best model, we also found the age class had statistically significant effect on parasites infection intensity in wild giant pandas (*X*^2^ = 18.47, *P* = 0.00035) (Fig. 3B). However, sex had no effect on the parasite infection intensity in wild giant pandas (*W* = 4269, *P* = 0.2991), although males appeared to have a slightly higher average infection intensity than females (Fig. 3A).

**Table 3:** Summary of GLMM results, evaluating the negative binomial distribution models responsible for explaining the parasites infection intensity and cortisol levels in wild giant pandas. Selected models are marked in bold

**Model**	**Parasites**		**Cortisol**	
	**AIC**	**Pr(>Chisq)**	**AIC**	**Pr(>Chisq)**
Null model	1997.8		2858.8	
Sex + age class + reproductive season + diet	1994.9	0.985	2816.2	0.233
**Age class + reproductive season + diet**	1993.6	0.002	**2814.4**	1.49E-09
Sex + reproductive season + diet	1992.9	0.326	2829.4	0.834
Sex + age class + diet	1992.9	<2.2E-16	2815.7	1.000
Sex + age class + reproductive season	2000.7	1.000	2848.5	1.000
Sex + age class	1999	0.179	2846.8	9.58E-06
Sex + diet	1991	<2.2E-16	2828.7	<2.2E-16
Reproductive season + diet	1991.9	1.000	2827.4	<2.2E-16
Sex + reproductive season	2000	0.296	2862.7	0.910
Sex	1998.7	0.290	2860.8	0.914
Age class	1997.8	0.042	2845.1	9.31E-06
Reproductive season	1999.1	1.000	2860.7	<2.2E-16
Diet	1989.9	<2.2E-16	2826.7	<2.2E-16
Sex × age class	2002.8	1.000	2849.8	1.000
Sex × reproductive season	1998.8	1.000	2864.4	1.000
Sex × diet	1990.4	<2.2E-16	2827.1	<2.2E-16
Age class × reproductive season	2000.9	1.000	2849	1.000
Age class × diet	1996.4	0.495	2818.3	1.000
**Reproductive season × diet**	**1979.2**	<2.2E-16	2827.3	<2.2E-16

The model with lowest AICc values (bold) were selected as the most parsimonious for our data analysis.

## Levels of metabolites of cortisol in faeces

**Figure 4:**
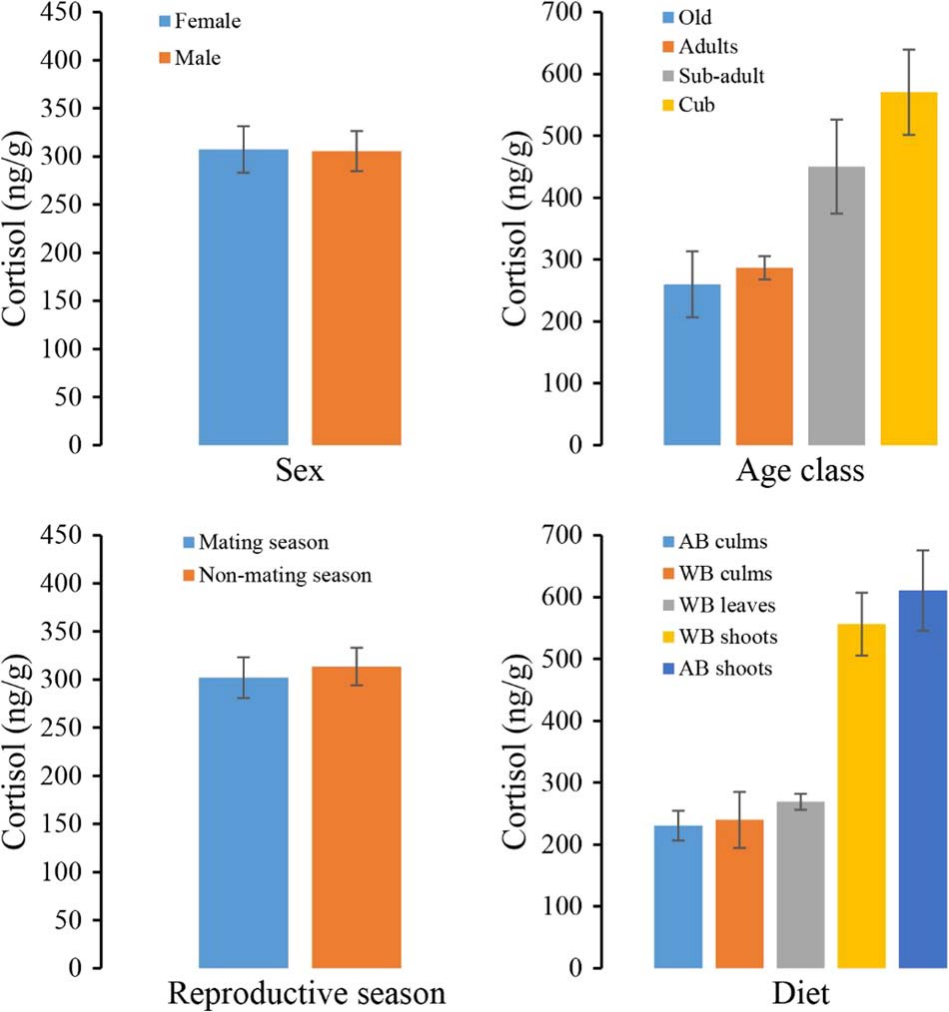
Variation in faecal cortisol levels in giant pandas by sex (A), age (B), reproductive season (C) and diet (D).

The best model showed that age class, reproductive season and diet affected cortisol metabolite levels in wild giant pandas (Table 3). The sex of giant pandas had no significant influence on the faecal cortisol levels (females, 307.27 ± 24.13 ng/g dry faeces; and males, 305.56 ± 20.85 ng/g dry faeces; *W* = 3175, *P* = 0.4825) (Fig. 4A). The faecal cortisol levels of cubs (570.63 ± 68.89 ng/g dry faeces) and sub-adults (450.40 ± 76.08 ng/g dry faeces) were higher than adult and old pandas (*X*^2^ = 20.35, *P* = 0.00014) (Fig. 4B). Although reproductive season was included in the best model, we failed to find any statistically significant effect of this variable on faecal cortisol levels in wild pandas (mating season, 302.13 ± 21.18 ng/g dry faeces; and non-mating season, 313.63 ± 19.53 ng/g dry faeces; *W* = 4898.5, *P* = 0.9209) (Fig. 4C). Peak cortisol levels were observed in the bamboo shoot-eating period (AB shoots, 610.8 ± 217.89 ng/g dry faeces; and WB shoots, 556.40 ± 50.81 ng/g dry faeces), which were significantly higher than the leaves and culms period (*W* = 6708.5, *P* < 0.0001) (Fig. 4D).

## Discussion

Stress responses exhibit seasonality in giant pandas, and the seasonal changes in parasite infection were consistent with the stress responses. Parasite infection intensities are the highest in May, when the cortisol levels were also at peak values. Surprisingly, we also found that the lowest protein-to-fibre ratio in the diets, which were occurred during the nutritional limited period in late winter and early spring, neither elevated the parasitic infections (i.e. high prevalence of *B. schroederi*) nor increased the cortisol levels. This seems to be inconsistent with the nutritional restriction hypothesis, which suggests that food and water shortages that act as stressors can elevate cortisol levels and parasitic infections in wild animals (Chapman *et al*., 2006; Behie and Pavelka, 2013; Hernandez *et al*., 2018).

Parasites and hosts coevolve, and the parasites often more rapidly evolve in comparison to their hosts, as they cycle through many generations during a single generation of the host (Anderson and May, 1982). In contrast to other mammals, giant pandas exclusively consume bamboo that has a particularly low nutritional content, and this may be the main reason for the seasonal change in parasite infection. Through the long period of coevolution, parasites may have adjusted many aspects of their life histories to adapt to the low-nutrient diet of giant pandas. Our study obtained a consistent result with those of previous studies, and the number of faecal parasite eggs was the highest during the bamboo shoot-eating period (Peng *et al*., 2017). We speculated that essential nutrients were needed for the growth and reproduction of parasites and might increase ovulation when pandas (hosts) used a high-quality diet, bamboo shoot (Coop and Kyriazakis, 2001). Furthermore, relative warm and wet habitats favour parasite egg and larval development in late spring and summer. In environmentally transmitted stages, the parasites eggs may be discharged with faeces and washed away by rainfall to infect other individuals through the polluted water (see Fig. 2B) (Gatcombe *et al*., 2010).

Although the results show that sex had no effect on the infection intensity of the parasites in wild giant pandas, we combined the results of individual identification and found that males were more likely to be infected than females. Pandas expand their home range during the bamboo shoot-eating period and mating season, which could increase encounters with the environmental transmission stages of parasites (Ostfeld *et al*., 2005). Additionally, the larger home range of males not only provides the chance to come into contacts with more conspecifics of the opposite sex but also increases the risk of acquiring contagious parasites (Jardine *et al*., 2014). However, females have smaller home ranges (Pan *et al*., 2001), which might reduce the chances of being exposed to parasites or maintaining high parasite loads. Similar to the infection patterns of raccoons (Jardine *et al*., 2014), cubs of giant pandas harboured the most parasites (the number of the *B. schroederi* eggs per unit mass of faeces) and might be easily susceptible to parasites because of their immature immune system. It is surprising that four old individuals had relatively lower parasite infection intensity than adults and cubs. We suggested that this might be caused by the seasonal sampling bias on the old individuals, because most of the faeces of the old ones were collected from October to April of the following year, when the parasite ovulation was significantly lower than the bamboo shoot period.

In this study, we found that the seasonal changes in cortisol levels in faeces were similar with the dynamic of parasite infection intensity. A high parasite load, might as a stressor, led to increased cortisol levels in giant pandas, and the cortisol levels might also influence the parasite load. Previous study indicated that low food availability was associated with increased faecal cortisol and parasite levels in primates (Chapman *et al*., 2006, 2007). Similar to parasite infection, we also suggested that the increased faecal cortisol levels were also related to the diets because low food availability could limit the nutrient intake. However, the cortisol levels of the pandas did not change in late winter and early spring, when the ratio of protein to fibre in the food was at the lowest value, but significantly increased from April to August, when the high protein-to-fibre ratio was found. Although the giant panda occupies different foraging niches in comparison to the sympatric wild boar and golden takin in the most time of the year, they show remarkable dietary competition with each other when consuming a highly preferred food, bamboo shoots, from May to August (Wei *et al*., 2017; Nie *et al*., 2019a). The wild boar, as the main competitor during the bamboo shoot-eating period, can not only consume bamboo shoots thoroughly but also destroy bamboo roots. The increased interspecific competition between the wild boar and giant panda might also cause increased intraspecific competition for the giant panda. Thus, we suggested that interspecific and intraspecific competition for bamboo shoots might be an important reason for the increase in cortisol levels during this period. Meanwhile, the elevated stress related to competition might also make the pandas more vulnerable to parasites.

The young pandas have higher cortisol level than the old ones indicated that they might face more pressures. We suggested that the exploration on their living environments might contribute this. We did not find the significant effects of sex and reproductive season on the cortisol levels of pandas. Our previous study showed that steroid hormone (e.g. testosterone) only increased in the short estrus period (usually one week), and went down to the baseline level immediately after that. As a large solitary species with no threats from predators, giant pandas often maintain a stable and low cortisol levels most of the time. Usually, only a few individuals would be in the reproductive status in the mating season. Therefore, at the whole mating season scale and the population level, the mean cortisol level did not show significant difference with that of other time.

## Conclusions

The seasonal dynamic of the cortisol levels and parasite load in faeces co-vary with the seasonal nutrient intake levels of the giant pandas, which concurrently arrived the peaks in the season when nutrition intake was high. Age class and seasonal food availability were the main factors affecting the parasite load and stress physiology in giant pandas. To the best of our knowledge, this is the first study to evaluate the influence of the sex, age, reproductive season and seasonal food availability on the parasitism and stress physiology in wild giant pandas. Our findings help to understand how environmental factors might influence the physiology, behaviour and health of wild pandas. More broadly, our study has implications for the conservation and management of this endangered species, not only for the wild populations but also the captive ones. For example, the effects of the seasonal food quality and availability limitation should be considered into the population monitoring of the wild pandas, especially for cubs and sub-adults. For the captive populations, high-nutrition diets should be controlled, under the premise of ensuring proper nutrition, to avoid high parasite load.

## Authors’ contributions

F.W., Y.N. and W.Z. conceived the ideas and designed methods; W.Z., K.G., Y.M., L.W. and Y.N. collected the samples; W.Z. performed nutritional and parasite infection analyses; W.Z., Y.M. and L.W. performed hormone and DNA analysis; W.Z. and Y.N. analysed data; W.Z., Y.N. and F.W. wrote the manuscript. All authors contributed critically to the drafts and gave final approval for publication.
